# Cyclic Conversions in the Nitrogen Cycle

**DOI:** 10.3389/fmicb.2021.622504

**Published:** 2021-03-24

**Authors:** Robbert Kleerebezem, Sebastian Lücker

**Affiliations:** ^1^Department of Biotechnology, Delft University of Technology, Delft, Netherlands; ^2^Department of Microbiology, IWWR, Radboud University, Nijmegen, Netherlands

**Keywords:** nitrification, anammox, thermodynamics, stoichiometry, denitrification

## Abstract

The cyclic nature of specific conversions in the nitrogen cycle imposes strict limitations to the conversions observed in nature and explains for example why anaerobic ammonium oxidation (anammox) bacteria can only use nitrite – and not nitrate – as electron acceptor in catabolism, and why nitrite is required as additional electron donor for inorganic carbon fixation in anabolism. Furthermore, the biochemistry involved in nitrite-dependent anaerobic methane oxidation excludes the feasibility of using nitrate as electron acceptor. Based on the cyclic nature of these nitrogen conversions, we propose two scenarios that may explain the ecological role of recently discovered complete ammonia-oxidizing (comammox) *Nitrospira* spp., some of which were initially found in a strongly oxygen limited environment: (i) comammox *Nitrospira* spp. may actually catalyze an anammox-like metabolism using a biochemistry similar to intra-oxic nitrite-dependent methane oxidation, or (ii) scavenge all available oxygen for ammonia activation and use nitrate as terminal electron acceptor. Both scenarios require the presence of the biochemical machinery for ammonia oxidation to nitrate, potentially explaining a specific ecological niche for the occurrence of comammox bacteria in nature.

## Introduction

Catabolic processes in chemotrophic microorganisms either rely on (organic) substrate fermentations or on oxidation of an electron donor with an external electron acceptor. Metabolic energy conservation in non-fermentative catabolic processes typically depends on the transfer of electrons from a reduced electron donor (e.g., organic carbon) to an electron carrier such as NAD under formation of NADH. NADH subsequently serves as electron donor for the respiratory chain where electrons are transferred through a series of respiratory protein complexes to a terminal electron acceptor such as molecular oxygen or oxidized nitrogen compounds like nitrite or nitrate. Metabolic energy available in these redox reactions is used for proton translocation across the cytoplasmic membrane, resulting in a proton motive force. An example process from the nitrogen cycle that is driven by respiration of nitrite or nitrate is heterotrophic denitrification (Strohm et al., 2007; Simon and Klotz, 2013). The versatility of electron carriers like NADH enable an unrestricted flexibility in the number of electrons donated and accepted in the catabolic reaction system, and different electron donor and electron acceptor reactions can be coupled directly.

Other conversions in the nitrogen cycle, primarily autotrophic nitrogen conversions, depend on more direct transfer of electrons from a donor to an acceptor in a dedicated respiratory system that does not involve electron carriers like NADH. In these so-called cyclic conversions electron transfer, the electron donor or an oxidized derivative thereof, directly reacts with the electron acceptor or an intermediate in the electron acceptor reaction. Due to the more direct reaction of an electron donor with and electron acceptor, cyclic conversions are restricted by tight stoichiometric dependencies that impose strict boundaries to a number of nitrogen conversions (Kartal et al., 2012).

In this manuscript two well-known cyclic conversions in the microbial nitrogen cycle are described and the specific consequences and restrictions associated with the cyclic nature of these conversions are identified. Based on these examples, the ecological implications for the recently discovered comammox metabolism are elaborated and discussed.

## Why Anaerobic Ammonium Oxidation With Nitrite, and Not Nitrate as Electron Acceptor?

The anammox process concerns the anaerobic oxidation of ammonium with nitrite as electron acceptor as discovered approximately 30 years ago (Strous et al., 1999a,b):

$$\begin{array}{l} 1\;\cdot \;NH_{4}^{+}+1\;\cdot \;NO_{2}^{-}\to 1\;\cdot \;N_{2}+2\;\cdot \;H_{2}O\\ \;△G^{0^{\prime }}\;=\;-362.8\;kJ\;\cdot \;reaction^{-1} \end{array}$$

All Gibbs free energy change values calculated are corrected for a pH of 7 (△*G*^0′^) and based on the Gibbs energy of formation values listed in Table 1. Microorganisms that are capable of using the anammox reaction for autotrophic growth depend on the oxidation of nitrite to nitrate for reduction of carbon dioxide to biomass precursors, presumably with ammonium as nitrogen source:

$$\begin{array}{l} 1\cdot CO_{2}+2.1\cdot NO_{2}^{-}+0.2\cdot NH_{4}^{+}+0.6\cdot H_{2}O\\ \;\to 1\cdot CH_{1.8}O_{0.5}N_{0.2}+2.1\cdot NO_{3}^{-}+0.2\cdot H^{+}\\ \;△G^{0^{\prime }}\;=\;+311.5\;kJ\cdot reaction^{-1} \end{array}$$

**TABLE 1:** Gibbs energy of formation values for the compounds involved in the reactions described.

compound	formula	phase	$G_{f}^{0}\left[kJ\cdot mol^{-1}\right]$
dinitrogen	*N_2*	g	0,0
nitric oxide	*NO*	g	86,6
nitrous oxide	*N_2O*	g	104,2
hydroxylamine	*NH*_2_*OH*	aq	−43,6
hydrazine	*N_2H_4*	g	159,2
ammonium	$NH_{4}^{+}$	aq	−79,4
nitrite	$NO_{2}^{-}$	aq	−32,2
nitrate	$NO_{3}^{-}$	aq	−111,3
carbon dioxide	*CO*_2_	g	−394,4
biomass	*CH*_1.8_*O*_0.5_*N*_0.2_	s	−67,0
methane	*CH_4*	g	−50,8
methanol	*CH_3OH*	aq	−175,4
oxygen	*O_2*	g	0,0
water	*H_2O*	aq	−237,2
proton	*H*^+^	aq	0,0
electron	*e*^−^	aq	0,0

The growth efficiency as reflected in the biomass yield per mole substrate has been measured to amount $1.30-1.70g/molNH_{4}^{+}$. Using a biomass yield value of $1.70g/molNH_{4}^{+}$, corresponding to $0.07Cmol/molNH_{4}^{+}$, the stoichiometry of anammox metabolism becomes:

$$\begin{array}{l} 1.00\cdot NH_{4}^{+}+1.13\cdot NO_{2}^{-}+0.07\cdot CO_{2}\to 0.99\cdot N_{2}\\ \;+0.15\cdot NO_{3}^{-}+0.07\cdot CH_{1.8}O_{0.5}N_{0.2}+1.93\cdot H_{2}O+0.01\cdot H^{+}\\ \;△G^{0^{\prime }}\;=\;-336.2\;kJ\cdot reaction^{-1} \end{array}$$

When expressed per mole of biomass formed, the Gibbs free energy change of the anammox process equals −4865*kJ*/*molX*. This value is comparable to the values for other autotrophic conversions that involve reversed electron transfer for carbon dioxide fixation (−3500*kJ*/*molX*, (Heijnen and Kleerebezem, 2010; Kleerebezem and Van Loosdrecht, 2010)), which suggests that the efficiency of the coupling of catabolism and anabolism in the anammox process is comparable to other autotrophic processes that require reversed electron transfer for carbon fixation.

**FIGURE 1:**
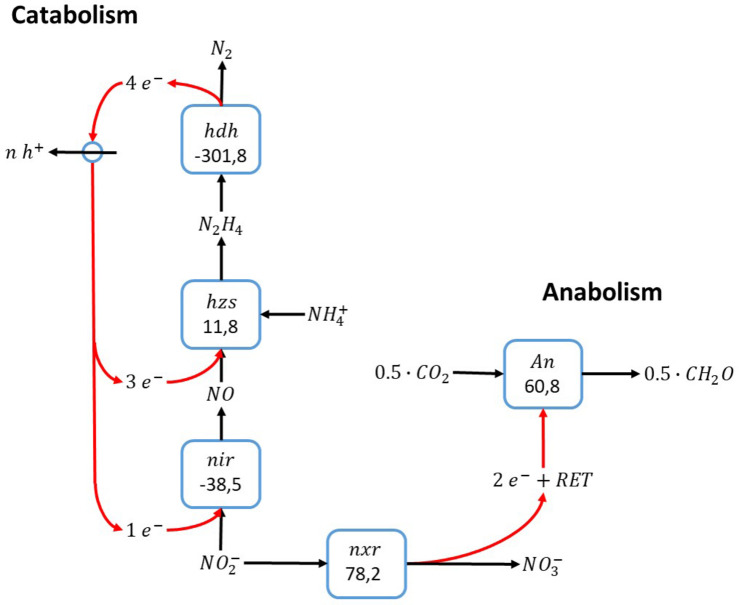
Anammox reaction stoichiometry, assuming ammonium activation with nitric oxide (Kartal et al., 2011). The individual blocks represent enzymatic steps (*hdh*, hydrazine dehydrogenase; *nir*, nitrite reductase; *hzs*, hydrazine synthase; *nxr*, nitrite oxidoreductase) and the corresponding Gibbs free energy change (△*G*^0′^) of the reaction corrected for a pH of 7. *RET* stands for reversed electron transfer.

The biochemistry of the anammox process as described by Kartal et al. (2011) is shown in Figure 1. In short: The electron acceptor in catabolism, nitrite, is reduced to nitric oxide by a nitrite reductase (*NiR*), and nitric oxide subsequently reacts with ammonium to form hydrazine. Energy-rich hydrazine is oxidized to dinitrogen gas and energy is harvested through formation of a proton motive force. Electrons obtained upon oxidation of hydrazine to dinitrogen gas are used for reducing nitrite to nitric oxide and the condensation of ammonium and nitric oxide to hydrazine. Carbon dioxide reduction to biomass, presumably with ammonium as nitrogen source, uses electrons obtained from nitrite oxidation to nitrate. Since the direct coupling of nitrite oxidation to nitrate and carbon dioxide reduction to biomass precursors is thermodynamically unfavorable, reversed electron transfer is required to drive anabolism. Herewith nitrite plays a peculiar double role in the anammox process: it serves as electron acceptor in catabolism and electron donor in anabolism.

The anammox catabolism is a typical example of a cyclic conversion. There is symmetry in the electron donor and acceptor reactions, where the three electrons gained upon oxidation of ammonium to dinitrogen gas equals the number of electrons required for nitrite reduction to dinitrogen gas. The prerequisite that ammonium in this reaction is activated by nitric oxide determines that ammonium needs to react with nitrite in a one-to-one ratio. In other words, at least one nitric oxide needs to be produced in the electron acceptor reaction to activate one ammonium.

This dependency of the anammox process on nitric oxide for ammonium activation also explains the incapacity of anammox bacteria to use ammonium as electron donor for anabolism: as each ammonium needs to be activated by one nitric oxide molecule, it is impossible to increase the ammonium to nitrite ratio to a value higher than one, which would be required if ammonium is used as anabolic electron donor for carbon dioxide reduction. The only remaining compound present in the system to serve as electron donor for carbon fixation is nitrite, which can be oxidized to nitrate. Indeed, growth of anammox is always associated with nitrate production according to a stoichiometry close to the metabolic reaction stoichiometry shown above.

The cyclic nature of the anammox process also explains directly that the potential oxidation of ammonium with nitrate under formation of dinitrogen gas according to

$$\begin{array}{l} 1\cdot NH_{4}^{+}+0.6\cdot NO_{3}^{-}\to 1.6\cdot N_{2}+1.8\cdot H_{2}O+0.4\cdot H^{+}\\ \;△G^{0^{\prime }}\;=\;-296.7\;kJ\cdot reaction^{-1} \end{array}$$

cannot be catalyzed with a biochemistry comparable to the scheme shown in Figure 1, because nitric oxide cannot be produced in the required 1:1 ratio with ammonium due to an unbalance in the number of electrons required to reduce nitrate ultimately to dinitrogen gas and electrons gained from ammonium oxidation. This is directly reflected in the ammonium to nitrate ratio of 1:0.6 in the reaction shown above.

A cyclic conversion that facilitates ammonium oxidation with nitrate as electron acceptor can be anticipated if not dinitrogen gas but nitrous oxide would be the end-product of the catabolic process:

$$\begin{array}{l} 1\cdot NH_{4}^{+}+1\cdot NO_{3}^{-}\to 1\cdot N_{2}O+2\cdot H_{2}O\\ \;△G^{0^{\prime }}\;=\;-179.5\;kJ\cdot reaction^{-1} \end{array}$$

Even though the Gibbs free energy change of this reaction is much less favorable than the original anammox reaction with nitrite as electron acceptor, there is no direct rational why this reaction would not occur. There is, however, another reason that makes this catabolic reaction unlikely to occur: In absence of nitrite there is no suitable electron donor that can be used to drive the reduction of carbon dioxide to biomass.

On the other hand, the cyclic nature of anammox catabolism allows the use of nitric oxide (NO) as electron acceptor in the process according to:

$$\begin{array}{l} 1\cdot NH_{4}^{+}+1.5\cdot NO\to 1.25\cdot N_{2}+1.5\cdot H_{2}O+1\cdot H^{+}\\ \;△G^{0^{\prime }}\;=\;-446.2\;kJ\cdot reaction^{-1} \end{array}$$

as has indeed been observed by Hu et al. (2019). In this case, the ammonium to nitric oxide ratio is smaller than 1, which implies that another (unknown) mechanism is available for NO reduction to dinitrogen gas (or ammonium) with electrons from hydrazine oxidation, besides the activation of ammonium like in the reaction scheme shown in Figure 1. One of the intriguing possibilities of the use of NO as electron acceptor is that it enables the use of hydrazine as very strong electron donor for carbon dioxide reduction to biomass precursors without the need of reversed electron transfer because anabolism becomes a thermodynamically favorable reaction:

$$\begin{array}{l} 1\cdot CO_{2}+1.05\cdot N_{2}H_{4}+0.2\cdot NH_{4}^{+}\to 1.05\cdot N_{2}\\ \;+1\cdot CH_{1.8}O_{0.5}N_{0.2}+1.5\cdot H_{2}O+0.2\cdot H^{+}\\ \;△G^{0^{\prime }}\;-187.6\;kJ\cdot reaction^{-1} \end{array}$$

This would exclude the need for anabolic nitrate production as experimentally demonstrated by Hu et al. (2019). Unfortunately, these authors did not report the impact on biomass yield in the process, which is expected to be significantly higher due to the absence of reversed electron transfer for carbon fixation (Kleerebezem and Van Loosdrecht, 2010).

A comparable increase in the energy content of the electrons needed in anabolism can also be achieved with nitrite as electron acceptor, provided that an efficient electron transfer mechanism between *nir* and *nxr* is available to drive nitrite disproportionation. This would then result in nitrite reduction to nitric oxide, combined with nitrite oxidation to nitrate as electron donor reaction as proposed by Hu et al. (2019). To which extent nitrite disproportionation is used in anammox to circumvent reversed electron transfer for carbon fixation remains unclear. The measured biomass yield values and the corresponding Gibbs free energy dissipation values for biomass production (see above) do suggest severe thermodynamic growth inefficiencies as typically associated with reversed electron transfer for carbon fixation.

## Why Direct Methane Oxidation With Nitrite, but Not Nitrate as Electron Acceptor?

A more recent breakthrough in the microbial nitrogen cycle is the experimental demonstration of methane oxidation with nitrite and nitrate as electron acceptor (Raghoebarsing et al., 2006):

$$\begin{array}{l} 1\cdot CH_{4}+2.67\cdot NO_{2}^{-}+2.67\cdot H^{+}\to 1\cdot CO_{2}+1.33\cdot N_{2}+3.33\cdot H_{2}O\\ \;△G^{0^{\prime }}\;=\;-941.8\;kJ\cdot reaction^{-1}\\ 1\cdot CH_{4}+1.6\cdot NO_{3}^{-}+1.6\cdot H^{+}\to 1\cdot CO_{2}+0.8\cdot N_{2}+2.8\cdot H_{2}O\\ \;△G^{0^{\prime }}\;=\;-765.7\;kJ\cdot reaction^{-1} \end{array}$$

**FIGURE 2:**
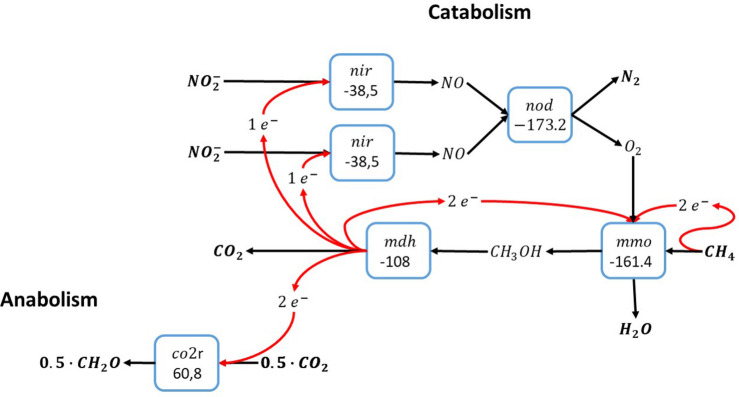
Methane oxidation with nitrite as electron acceptor via anaerobic production of molecular oxygen (Raghoebarsing et al., 2006). The individual blocks represent enzymatic steps (*nod*, nitric oxide dismutase; *nir*, nitrite reductase; *mmo*, methane monooxygenase; *mdh*, methanol dehydrogenase *co2r*, CO_2_ fixation); and the corresponding Gibbs free energy change (△*G*^0′^) of the reactions corrected for a pH of 7.

Methane oxidation with nitrite relies on the intracellular production of molecular oxygen from nitric oxide by a nitric oxide dismutase. Since the *Candidatus* Methylomirabilis bacteria that catalyze nitrite-dependent anaerobic methane oxidation use the particulate methane monooxygenase, which requires one molecule of molecular oxygen per methane, the minimum number of nitrite molecules reduced per methane is two (see Figure 2). For methane oxidation with nitrite more than two nitrite molecules are required to accept the electrons gained from methane oxidation. Consequently, the remaining electrons from methane oxidation can either be used for inorganic carbon conversion to biomass precursors (Rasigraf et al., 2014) as shown in Figure 2, or for more catabolic reduction of nitrite. Overall, there is no reason why methane oxidation with nitrite cannot be catalyzed by a single microorganism, as has indeed been demonstrated experimentally (Raghoebarsing et al., 2006; Ettwig et al., 2010).

The situation is different for methane oxidation with nitrate (Raghoebarsing et al., 2006; Arshad et al., 2015; Welte et al., 2016; Stultiens et al., 2019). In this catabolic system the boundary condition that at least two oxidized nitrogen compounds are required in order to produce one molecular oxygen suggests that in the overall reaction 10 electrons are accepted per oxygen produced. Evidently, this exceeds the eight electrons gained per methane oxidized. This suggests directly that using the biochemistry proposed, there cannot be one microorganism that is capable of catalyzing methane oxidation with nitrate as electron acceptor, as is indeed observed in experiments. Instead, it has been demonstrated that methane oxidation with nitrate can be catalyzed by *Methanoperedens* archaea using the reversed methanogenesis pathway under formation of nitrite or ammonium. This provides the ecological niche for coexistence with either anammox or *Candidatus* Methylomirabilis to establish full conversion of methane and nitrate to dinitrogen gas and carbon dioxide (Stultiens et al., 2019).

## Is Comammox a Cyclic Conversion?

Until recently aerobic microbial ammonia oxidation to nitrate (nitrification) was regarded as a process catalyzed by two distinct groups of microorganisms that both take care of a distinct part of the process: ammonia oxidizing bacteria (or archaea) oxidize ammonia to nitrite, and nitrite oxidizing bacteria oxidize nitrite to nitrate. Both conversions are aerobic conversions. The reason why this pathway is segregated in two different types of microorganisms has been the topic of intense debate, but to date no convincing hypothesis has been proposed that explains the advantage of pathway segregation. It has been proposed that longer catabolic pathways provide a competitive advantage over short pathways in case of severe substrate limitation because it allows for maximization of the amount of energy that can be harvested per unit of substrate (i.e., ammonia). This maximization of the biomass yield strategy was proposed to enable the enrichment of complete ammonia oxidizing (comammox) microorganisms in case of slow growing substrate-limited systems such as biofilms (Costa et al., 2006).

Recently, convincing proof was presented that ammonia oxidation to nitrate is not necessarily catalyzed by a tandem of an ammonia oxidizer and a nitrite oxidizer, and specific *Nitrospira* species do have the capacity to oxidize ammonia to nitrate (Daims et al., 2015; van Kessel et al., 2015). *Nitrospira* are primarily known as aerobic chemolithoautotrophic nitrite oxidizing bacteria, with the capacity to conduct a number of other functions such as respiration of simple organic carbon molecules or hydrogen (Daims and Wagner, 2018).

Although comammox *Nitrospira* have been detected in a range of natural and engineered environments, their ecological role in many of these systems remains unclear (Daims et al., 2015; van Kessel et al., 2015; Palomo et al., 2016; Camejo et al., 2017; Xia et al., 2018; Li et al., 2019; Poghosyan et al., 2019; Cotto et al., 2020). Some of the first comammox bacteria were initially identified by van Kessel et al. (2015) in a bioreactor fed with effluent water from a recirculating aquaculture system, amended with low concentrations of ammonium, nitrite and nitrate. No molecular oxygen was supplied and therefore only oxygen traces in gases and medium supplied may have entered the bioreactor. Compared to oxygen, an overdose of oxidized nitrogen compounds (nitrite and nitrate) were supplied as electron acceptors to the system. Unfortunately, no mass balance measurements are available for the nitrogen compounds in the process, so the quantitative ecological role of comammox *Nitrospira* spp. could not be identified. Still, the system described can be considered a strongly oxygen limited system.

Recent studies that aimed for *in situ* detection of comammox *Nitrospira* species have also observed them in significant numbers in systems that are characterized by localized oxygen limitation, such as biofilm systems (Roots et al., 2019). Furthermore, ammonium limitation and long solid retention times seem to favor enrichment of comammox (Kits et al., 2017; Cotto et al., 2020). Here, we focus on the observation by van Kessel at al. (2015) that comammox *Nitrospira* spp. were identified as dominant community members in a severely oxygen limited enrichment culture grown on a mixture of ammonium, nitrite, and nitrate.

Despite the oxygen limitation, comammox *Nitrospira* spp. constituted a considerable fraction of the biomass in this system. It is counterintuitive to assume that oxygen limitation provides a strong competitive advantage for comammox bacteria because the aerobic ammonia oxidation to nitrate *maximizes* oxygen uptake per mole of ammonium. At strongly oxygen limited conditions *minimization* of the stoichiometric needs for oxygen would provide the microorganisms involved with a competitive advantage. However, this would make the unique capacity of comammox to oxidize ammonia to nitrate nothing more than a coincidence. Thus, this raises the question why comammox has been found in this strongly oxygen limited ecosystem and suggests that there must be an intrinsic competitive advantage of having the full catabolic pathway for ammonia oxidation to nitrate.

Overall, it is proven that the specific *Nitrospira* species do have the capacity to catalyze the oxidation of ammonia to nitrate, but to which extent this is the metabolic trait that provides them with a competitive advantage over canonical aerobic autotrophic ammonia oxidisers that produce nitrite remains unclear. Below we will propose two cyclic ammonia oxidation processes that both require the microorganisms involved to possess the capacity to oxidize ammonia to nitrate, and that would be a satisfying explanation why some comammox bacteria have first been encountered in a strongly oxygen limited ecosystem.

## Scenario 1: Is Comammox Functionally Equivalent to Anammox in Anaerobic Conditions?

The first pathway that may explain the in-situ activity of comammox bacteria (i) does not require externally supplied oxygen, and (ii) does require the full aerobic ammonia oxidation pathway to nitrate for sustaining the process. The conversion route proposed is based on a combination of the anammox process and the intra-oxic methane oxidation pathway with nitrite as electron acceptor as both described above.

**FIGURE 3:**
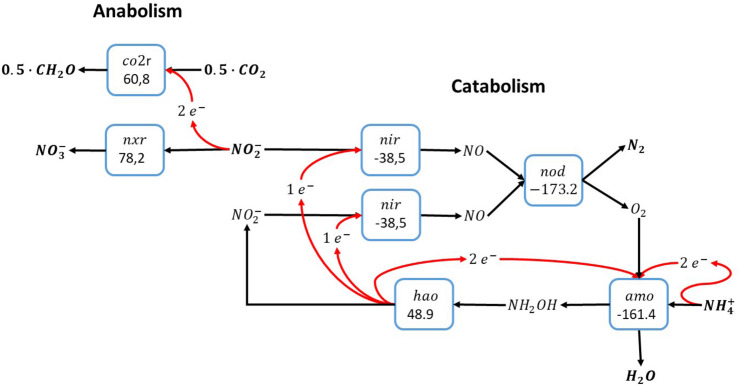
Ammonia oxidation with nitrite as electron acceptor via intracellular production of molecular oxygen (Raghoebarsing et al., 2006). This overall reaction is equivalent to the anammox reaction as shown in Figure 1. The individual blocks represent enzymatic steps (*nod*, nitric oxide dismutase; *nir*, nitrite reductase; *amo*, ammonia monooxygenase; *hao*, hydroxylamine dehydrogenase; *nxr*, nitrite oxidoreductase; *co2r*, CO_2_ fixation) and the corresponding Gibbs free energy change of the reactions corrected for a pH of 7 (△*G*^0′^).

An alternative pathway for the anammox catabolism as shown in Figure 1 involves the use of reactive oxygen instead of NO for the activation of ammonia, as shown for methane oxidation in Figure 2. Molecular oxygen would be produced via nitric oxide dismutation as proposed for anaerobic methane oxidation by *Candidatus* Methylomirabilis oxyfera. As opposed to anaerobic methane oxidation, the catabolic reaction shown in Figure 3 concerns a perfect cyclic conversion since two of the electrons gained upon oxidation of ammonia to nitrite ($6emol/molNH_{4}^{+}$) are required to reduce two nitrite to nitric oxide, with subsequent dismutation to dinitrogen gas and molecular oxygen, while the remaining four electrons would be required for the reduction of O_2_ at the ammonia monooxygenase. The resulting catabolic reaction equals anammox catabolism described before.

The cyclic nature of this anammox-like catabolism via nitric oxide dismutase imposes the necessity of an alternative electron donor for reduction of carbon dioxide to organic biomass precursors, like in anammox. Ammonia cannot be used as electron donor due to the cyclic properties of this catabolism, and the only electron donor available in the autotrophic system is nitrite, which is oxidized to nitrate. As a consequence, the metabolism proposed here requires the biochemical machinery for ammonia oxidation to nitrite in the catabolic system, and nitrite oxidation to nitrate in the anabolic system. In absence of the full oxidation pathway of ammonia to nitrate, this metabolic system cannot exist. Functionally, the overall pathway proposed here is fully equivalent to anammox metabolism as originally described by Strous et al. (1999a).

With regard to the experiments conducted by van Kessel et al. (2015), the reaction proposed here can pose an alternative explanation for the observations in the ^15^N-ammonium labeling experiments. Activity assays with anammox bacteria and a mixture of ^15^N-labeled ammonium and unlabeled nitrite typically result in the production of ^29^N_2_. However, upon partial ammonia oxidation to nitrite as shown in Figure 3, also ^30^N_2_ will be produced, which is in line with the data shown in the paper. Contrastingly, the production ^30^N_2_ using the comammox metabolism is hard to understand if nitrate is produced from ammonia, since anammox is incapable of using nitrate as electron acceptor. However, the reaction requires the presence of a nitric oxide dismutase as has been proposed for *Ca*. M. oxyfera (Ettwig et al., 2010) that could thus far not be identified in any comammox *Nitrospira* genome.

## Scenario 2: Is Comammox Capable of Ammonia Oxidation With Combined Use of Oxygen and Nitrate as Electron Acceptors in Oxygen Limited Conditions?

The second pathway we propose is based on the observation that these comammox bacteria have been identified in a system that, while characterized by severe oxygen limitation, might still have received minor amounts of oxygen (van Kessel et al., 2015).

It is counterintuitive that conditions of severe oxygen limitation would select for an ammonia oxidizing microorganism that requires *more* oxygen than canonical ammonia oxidation to nitrite. Under oxygen limiting conditions one would expect the occurrence of metabolic pathways that minimize oxygen requirements. The minimum amount of oxygen required per unit of ammonia evidently is one, due to the consumption of O_2_ by the ammonia monooxygenase. Due to this four-electron reduction of oxygen, the oxidation of ammonia to nitrite yields two electrons per mole ammonia:

$$\begin{array}{l} 1\cdot NH_{4}^{+}+1\cdot O_{2}\to 1\cdot NO_{2}^{-}+4\cdot H^{+}+2\cdot e^{-}\\ \;△G^{0^{\prime }}\;=\;-112.5\;kJ\cdot reaction^{-1} \end{array}$$

Under aerobic conditions the electrons will be accepted by oxygen, but we propose that in case of oxygen limitation nitrate may serve as alternative electron acceptor, with the concomitant formation of nitrite:

$$\begin{array}{l} 1\cdot NO_{3}^{-}+2\cdot H^{+}+2\cdot e^{-}\to 1\cdot NO_{2}^{-}+1\cdot H_{2}O\\ \;△G^{0^{\prime }}\;=\;-78.2\;kJ\cdot reaction^{-1} \end{array}$$

**FIGURE 4:**
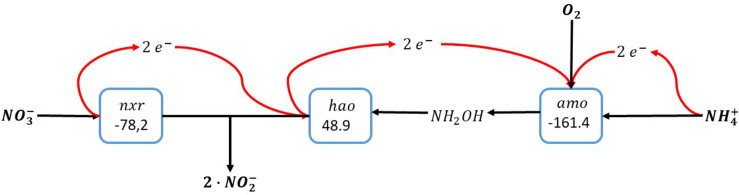
Ammonia oxidation with combined use of oxygen and nitrate as electron acceptors. The individual blocks represent enzymatic steps (*nxr*, nitrite oxidoreductase; *amo*, ammonia monooxygenase; *hao*, hydroxylamine dehydrogenase), and the corresponding Gibbs free energy change (△*G*^0′^) of the reactions corrected for a pH of 7.

Overall, this results in the conversion of ammonia with oxygen and nitrate, forming two nitrite in a so-called nitrite comproportionation reaction (Figure 4):

$$\begin{array}{l} 1\cdot NH_{4}^{+}+1\cdot O_{2}+1\cdot NO_{3}^{-}\to 2\cdot NO_{2}^{-}+1\cdot H_{2}O+2\cdot H^{+}\\ \;△G^{0^{\prime }}\;=\;-190.7\;kJ\cdot reaction^{-1} \end{array}$$

This pathway implies that the nitrite oxidoreductase (*nxr*) is reversible: In case of severe oxygen limitation nitrate can be reduced to nitrite in order to minimize the oxygen requirements for ammonia oxidation. Importantly, this reversibility of the nitrite oxidoreductase has been demonstrated for *Nitrospira moscoviensis*, which was able to couple formate oxidation to nitrate reduction under anaerobic conditions (Koch et al., 2015). In case of oxygen excess, nitrite is oxidized to nitrate facilitating the complete conversion of ammonia to nitrate. As in this reaction scheme the oxidation of ammonia is not directly dependent on products formed during the electron accepting reaction as is the case in the intra-oxic pathway described above, the electrons from ammonia oxidation here can also directly be used for carbon dioxide reduction to biomass precursors.

As described for the nitric oxide dismutase-based anammox-like pathway, the formation of ^30^N_2_ from ^15^N labeled ammonium as reported by van Kessel et al. (2015) can readily be explained by this pathway, as here the ^15^N-labeled nitrite formed by comammox *Nitrospira* will serve as substrate for anammox.

## Discussion

### Cyclic Conversions and the Microbial Nitrogen Cycle

The biochemistry-induced cyclic nature of some nitrogen conversions provides clear limits to the catalytic capacity observed in nature. Why anammox has been found only with nitrite as electron acceptor and not nitrate can readily be explained by the biochemistry of the anammox catabolism. Furthermore, also why anammox uses nitrite as electron donor in anabolism for carbon dioxide reduction to biomass is a direct resultant from the cyclic nature of anammox catabolism. Similarly, the biochemistry of anaerobic nitrite-dependent methane oxidation proposed for *Candidatus* Methylomirabilis oxyfera excludes the direct catabolic use of nitrate as electron acceptor for methane oxidation.

In this paper we have elaborated the consequences of the cyclic nature of nitrogen conversions on the potential ecological role of comammox *Nitrospira* under oxygen-limited conditions. Considering the biochemistry of the known nitrogen conversions, two alternative pathways are proposed to provide an ecological niche for comammox *Nitrospira*: an intra-oxic anammox-like metabolism using nitric oxide dismutase, or nitrite comproportionation, the oxidation of ammonia with both oxygen and nitrate as electron acceptors. Both pathways require the full enzymatic machinery for ammonia oxidation to nitrate in order to proceed, which adds to the plausibility of the pathways proposed. On the other hand, no direct evidence is available to date that either of the two pathways proposed actually occurs in microbial ecosystems, and no genomic evidence supports the existence of a nitric oxide dismutating enzyme system in *Nitrospira*. Furthermore, arguments related to the bioenergetic growth efficiency and kinetic properties of the processes proposed that may determine ecological niches for specific conversions are excluded from the discussion (Gonzalez-Cabaleiro et al., 2019; Kreft et al., 2020).

### Microbial Ecosystems Aim for Thermodynamic Equilibrium

In absence of severe gradients in time or space, one may speculate that microbial ecosystems aim for maximization of the amount of chemical energy that is harvested, provided that the adequate biochemistry is available to do so, and sufficient time is available for the microbes to establish. In other words, microbial ecosystems thrive for a thermodynamic state as close as possible to thermodynamic equilibrium. For example, in absence of external electron acceptors, organic carbon rich environments aim for the production of methane containing biogas because methane is the organic carbon with the lowest energy content per electron ($△G_{e}^{0^{\prime }}$) of all organic compounds (Kleerebezem and Van Loosdrecht, 2010). Full conversion of organic carbon to methane can therefore be regarded as the global thermodynamic optimum, whereas the production of intermediate compounds in the process represent local optima. If the thermodynamically most favorable solution for a specific ecosystem does not occur – such as the oxidation of ammonium with nitrate to dinitrogen gas - this implies that either the biochemistry to catalyze this overall reaction does not exist, or has not been discovered yet.

In an oxygen limited environment with non-limiting ammonium as well as oxidized nitrogen compounds (nitrite and nitrate) available, as described by van Kessel et al. (2015), the complete oxidation of ammonia to nitrate with oxygen would thermodynamically be one of the least favorable reactions to catalyze when normalized to one mole of oxygen:

$$\begin{array}{l} 0.5\cdot NH_{4}^{+}+1\cdot O_{2}\to 0.5\cdot NO_{3}^{-}+0.5\cdot H_{2}O+1\cdot H^{+}\\ \;△G^{0^{\prime }}\;=\;-174.5\;kJ\cdot mol\;O_{2}{}^{-1} \end{array}$$

In comparison, the combined activity of aerobic ammonia oxidation to nitrite by for example *Nitrosomonas* spp. combined with the anammox reaction (Figure 1) provides a significantly higher Gibbs free energy yield:

$$\begin{array}{l} 1.33\cdot NH_{4}^{+}+1\cdot O_{2}\to 0.67\cdot N_{2}+1.33\cdot H^{+}+2\cdot H_{2}O\\ \;△G^{0^{\prime }}\;=\;-421.7\;kJ\cdot mol\;O_{2}{}^{-1} \end{array}$$

Still, in the presence of nitrate in the medium, and assuming the conversion of part of the ammonium as proposed in Scenario 2 (Figure 4) combined with the oxygen independent anammox catabolism, the amount of energy that is harvested is much higher:

$$\begin{array}{l} 3\cdot NH_{4}^{+}+1\cdot O_{2}+1\cdot NO_{3}^{-}\to 2\cdot N_{2}+2\cdot H^{+}+5\cdot H_{2}O\\ \;△G^{0^{\prime }}\;=\;-916.3\;kJ\cdot reaction^{-1} \end{array}$$

In an oxygen limited environment where nitrite, nitrate and non-limiting amounts of ammonium are available, maximization of the conversion of ammonium for the production of dinitrogen gas with all electron acceptors available is the global thermodynamic optimum of the reaction network. However, when assuming that no biochemistry for direct ammonium oxidation with nitrate to dinitrogen gas is available in nature, the process described in scenario 2 will maximize the Gibbs energy production per unit of oxygen supplied. Evidently, this provides no evidence that no other local thermodynamic optimum may prevail, but it is tempting to speculate and study to which extent nature and evolution are optimized with the objective to maximize energy production in mind.

### Implications for Biofilms and Cell Aggregates

The potential existence of the metabolic pathways proposed here may shed new light on observations made with biofilm systems consisting of aerobic ammonia oxidizing bacteria in the outer layers of the biofilm, and anammox bacteria in the anaerobic core. In these biofilm systems *Nitrospira* is frequently encountered at the aerobic-anaerobic interface (Vlaeminck et al., 2010; Winkler et al., 2012). Traditionally, *Nitrospira* has been associated with aerobic nitrite oxidation to nitrate, and nitrate is not perceived to play a role in the overall conversion of ammonium to dinitrogen gas. Consequently, the appearance of *Nitrospira* in these systems is believed to negatively affect process performance, as it takes away nitrite from anammox and therewith decreases the nitrogen removal efficiency.

The catabolic reactions proposed here in scenarios 1 and 2 provide alternative, mechanistically plausible explanations for the occurrence of *Nitrospira* at the aerobic-anaerobic interface, assuming these have the enzymatic machinery for complete nitrification. According to scenario 1, oxygen may serve as intracellular intermediate in catalyzing anaerobic ammonia oxidation via nitric oxide dismutation, and scenario 2 proposes that at severe oxygen limitation, the use of nitrate besides oxygen in ammonia oxidation provides a competitive advantage over fully aerobic ammonia oxidation to nitrite. At more elevated oxygen concentrations closer to the bulk liquid in the biofilm, aerobic processes will prevail, whereas the anammox metabolism likely dominates at more reduced redox conditions in true absence of oxygen. It is furthermore plausible that when these biofilms are periodically exposed to elevated oxygen concentrations, the comammox *Nitrospira* in the system catalyze the full oxidation of ammonia to nitrate. However, these *Nitrospira* have the advantage that when they compete with canonical ammonia oxidizers for their substrate, they can also return to their core business, nitrite oxidation to nitrate. This may explain why the maximum nitrite oxidation capacity in these systems as determined at elevated dissolved oxygen concentrations usually is much higher than expected in these biofilm systems (Winkler et al., 2012).

In this manuscript we propose that the metabolic versatility of comammox *Nitrospira* spp. may be higher than described to date. The work of Kits et al. (2017) suggests that comammox *Nitrospira* spp. can in specific conditions be an effective competitor for two-step nitrification. In particular, the high affinity for ammonia together with the increased biomass yield will provide a competitive advantage over canonical ammonia oxidizing microorganisms under conditions of severe ammonium limitation. To which extent severe oxygen limitation provides a similar ecological niche for full oxidation of ammonia to nitrate is unclear, but seems unlikely. Here, we describe that instead of catalyzing ammonia oxidation to nitrate, oxygen limitation may provide an ecological niche for a nitric oxide dismutation-driven anammox-like metabolism, or ammonia oxidation with the combined use of nitrate and oxygen as electron acceptors. Future experiments under oxygen limiting conditions will need to clarify if these conditions specifically enrich for comammox *Nitrospira*, and what their ecological niche is in these kinds of environments.

## Data Availability Statement

The original contributions presented in the study are included in the article/supplementary material, further inquiries can be directed to the corresponding author.

## Author Contributions

All authors listed have made a substantial, direct, and intellectual contribution to the work and approved it for publication.

## Conflict of Interest

The authors declare that the research was conducted in the absence of any commercial or financial relationships that could be construed as a potential conflict of interest.
